# A Review of Exosomal Isolation Methods: Is Size Exclusion Chromatography the Best Option?

**DOI:** 10.3390/ijms21186466

**Published:** 2020-09-04

**Authors:** Karim Sidhom, Patience O. Obi, Ayesha Saleem

**Affiliations:** 1 Max Rady College of Medicine, Rady Faculty of Health Sciences, University of Manitoba, Winnipeg, MB R3E 3P5, Canada; sidhomk@myumanitoba.ca; 2 Diabetes Research Envisioned and Accomplished in Manitoba (DREAM) Research Theme of CHRIM, Winnipeg, MB R3E 3P4, Canada; obip@myumanitoba.ca; 3 Biology of Breathing Research Theme of CHRIM, Winnipeg, MB R3E 3P4, Canada; 4 Children’s Hospital Research Institute of Manitoba (CHRIM), Winnipeg, MB R3E 3P4, Canada; 5 Applied Health Sciences, Faculty of Graduate Studies, University of Manitoba, Winnipeg, MB R3T 2N2, Canada; 6 Faculty of Kinesiology and Recreation Management, University of Manitoba, Winnipeg, MB R3T 2N2, Canada

**Keywords:** extracellular vesicles, exosomes, microvesicles, differential ultracentrifugation, poly-ethylene glycol, immunoaffinity capture, microfluidics, size-exclusion chromatography

## Abstract

Extracellular vesicles (EVs) are membranous vesicles secreted by both prokaryotic and eukaryotic cells and play a vital role in intercellular communication. EVs are classified into several subtypes based on their origin, physical characteristics, and biomolecular makeup. Exosomes, a subtype of EVs, are released by the fusion of multivesicular bodies (MVB) with the plasma membrane of the cell. Several methods have been described in literature to isolate exosomes from biofluids including blood, urine, milk, and cell culture media, among others. While differential ultracentrifugation (dUC) has been widely used to isolate exosomes, other techniques including ultrafiltration, precipitating agents such as poly-ethylene glycol (PEG), immunoaffinity capture, microfluidics, and size-exclusion chromatography (SEC) have emerged as credible alternatives with pros and cons associated with each. In this review, we provide a summary of commonly used exosomal isolation techniques with a focus on SEC as an ideal methodology. We evaluate the efficacy of SEC to isolate exosomes from an array of biological fluids, with a particular focus on its application to adipose tissue-derived exosomes. We argue that exosomes isolated via SEC are relatively pure and functional, and that this methodology is reproducible, scalable, inexpensive, and does not require specialized equipment or user expertise. However, it must be noted that while SEC is a good candidate method to isolate exosomes, direct comparative studies are required to support this conclusion.

## 1. Introduction

**Figure 1:**
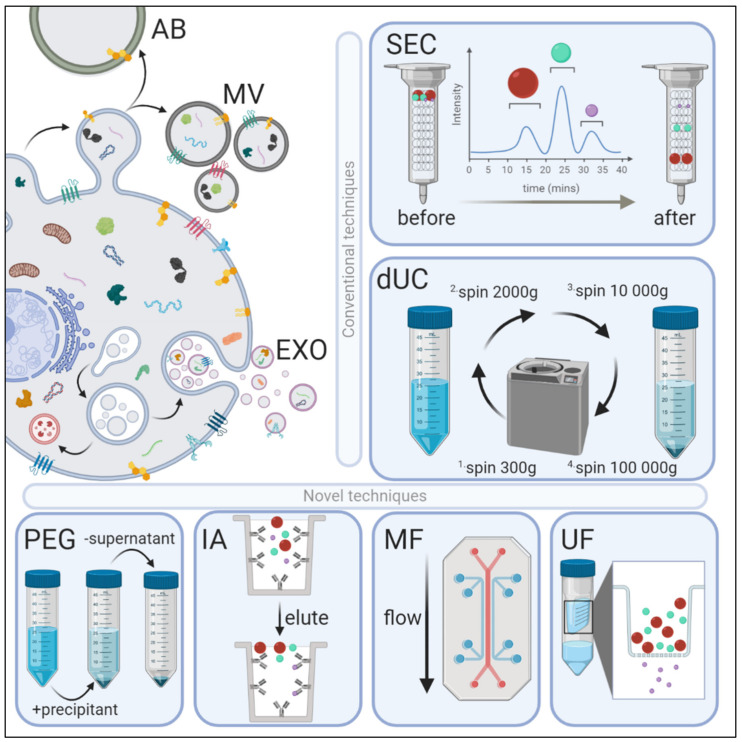
Extracellular vesicle (EV) biogenesis, subpopulations, and conventional and novel methods of exosome isolation. EVs are categorized into three main types depending on their site of origin, density, expression of markers, and/or size. Apoptotic bodies (AB) are released by the blebbing of an apoptotic cell membrane (500–5000 nm); microvesicles (MV) are shed from the outward budding of the plasma membrane (100–1000 nm); and exosomes (EXO) are formed when multivesicular bodies fuse to the plasma membrane and release intraluminal vesicles (30–150 nm). EVs have variable: (1) protein expression profiles—EXOs are enriched with Fliotillin-1, ALIX, TSG101, CD81, CD63 and CD9 proteins, whereas MVs preferentially express MMP2 and ARF6; (2) lipidomic profiles—MVs are enriched with phosphatidylserine and cholesterol, EXOs with sphingomyelin and ceramide, and ABs by phosphatidylserine; and (3) distinct genomic and transcriptomic luminal cargo. Conventional methods of EV isolation include size-exclusion chromatography (SEC) and differential ultracentrifugation (dUC). SEC uses biofluids as a mobile phase against a porous stationary phase to differentially elute molecules with an inverse speed relation to their size—in other words, larger particles will elute first, followed by smaller vesicles that will enter and flow through the pores resulting in a longer path and thus increased elution time. dUC relies on the separation of EV subpopulations via gradually higher acceleration rates. More novel exosomal techniques also exist. Poly-ethylene glycol (PEG)-based precipitation uses a solution to facilitate a polymer-entrapped vesicle aggregate in large numbers. Immunoaffinity (IA) capture uses antibodies targeted against exosomal surface proteins to isolate specific vesicle population. Microfluidics (MF) technology uses chips with specific antibody-mediated binding to capture exosomes efficiently. Ultrafiltration (UF) is dependent on a filter of specific pore size that creates a vesicle-rich filtrate specific to the desired size.

Extracellular vesicles (EVs) can be largely divided into three main subtypes: apoptotic bodies, microvesicles, and exosomes, and are classified based on their cellular origin, physiochemical, and biomolecular properties [1] (Figure 1). The largest of these EVs, apoptotic bodies arise from the outward blebbing of an apoptotic cell membrane, resulting in phosphatidylserine-rich vesicles 500–5000 nm in diameter. Microvesicles originate as particles shedding from the plasma membrane and are enriched with phosphatidylserine and cholesterol, and typically are 100–1000 nm in diameter. Exosomes are the smallest EVs (30–150 nm) and are formed by the exocytosis of multivesicular bodies (MVBs) liberating intraluminal vesicles upon fusion with the plasma membrane [2]. The biogenesis of these intraluminal vesicles occurs through endosomal sorting complexes required for transport (ESCRT) or a soluble N-ethylmaleimide sensitive factor (NSF) attachment protein receptor (SNARE)-based system. Exosomal membranes are characterized by the presence of specific lipid species: cholesterol, sphingomyelin, ceramide, and phosphatidylserine, some of which can be used to distinguish them from liposomes [3,4]. Exosomes represent an evolutionarily conserved mode of communication [5,6] and serve as critical mediators of intercellular communication and potentiate organ cross-talk.

Exosomes were first discovered in 1983 in two studies published within a week of each other, as endosomal vesicles containing transferrin receptors that were secreted from maturing reticulocytes [7,8]. The presence of exosomes was relatively ignored (classified as ‘cellular dust’) until interest was revived with discoveries demonstrating their capacity to serve as mediators of intercellular communication [9], potential drug delivery vectors [10], and bio-markers of various chronic and acute diseases [11,12,13,14,15]. Exosomes are a snapshot of the parent cell that produces them, and upon uptake by the recipient cell, can modify cell function by virtue of their enclosed cargo. Exosomal cargo can include proteins, DNA species (mitochondrial DNA and nuclear DNA), RNA species (mRNA, microRNA, and lncRNA), lipids, and metabolites. Exosomal cargo can change depending on the cellular milieu, cell of origin, and even the exosome preparation technique [16].

Exosome research is plagued with the use of inconsistent isolation methodologies, nomenclature, and a lack of standardized data acquisition and analysis strategies. This limits the interpretation of the research conducted on exosomes [17]. Furthermore, due to the differential protein constitution of exosomes, using conventional immuno-marking methodologies to isolate them is challenging, as not all exosomes express the same classical protein markers, nor are all established markers exclusive to exosomes, as they have been found in other subtypes of EVs as well. Indeed, the International Society for Extracellular Vesicles (ISEV) endorsed use of a new standardized nomenclature unless researchers can reliably establish the endosomal origin of their exosomal preparations. The Minimal Information for Studies of Extracellular Vesicles (MISEV) 2018 guidelines recommend characterization of EVs according to size [small EVs (sEVs < 200 nm); medium/large EVs (m/lEV > 200 nm)], density [low, medium, high], biochemical composition [e.g., CD81^+^/CD63^+^ EVs], or cell of origin [e.g., oncosomes, hypoxic EVs, etc.] [18]. Irrespective of nomenclature, exosomes and other EVs have been isolated by a number of techniques such as differential ultracentrifugation, size-exclusion chromatography, ultrafiltration, polyethylene glycol-based precipitation, immunoaffinity capture, or by using microfluidics [1,19]. Each method of isolation has inherent advantages and disadvantages, and provides a differential yield of exosomes that may be contaminated with EV subtypes and/or humoral protein aggregates. Here, we review the commonly used methods of exosome isolation, with a focus on size-exclusion chromatography (Figure 1, Table 1 and Table 2).

## 2. Methods of Exosome Isolation

### 2.1. Differential Ultracentrifugation (dUC)

This technique relies on the sequential separation of particles by sedimentation dependent on their size and density using a series of centrifugal forces and duration [50]. dUC can isolate relatively pure populations of exosomes and is generally considered to be the gold standard technique for exosomal isolation [51]. This extraction capacity is determined by a combination of factors as summarized in Table 3 [52]. Although contamination from non-exosome related proteins in the isolated pellet from dUC is low, lipoprotein particles of similar density tend to precipitate with the final pellet.

The dUC procedure begins with a number of cleaning spin steps designed to remove cells, cellular debris, apoptotic bodies, and microvesicles [19]. This is done by gradually separating the pellet and supernatant at increasing speeds: 300–400× *g* for 10 min, then 2000× *g*, and finally 10,000× *g*, to isolate a supernatant containing a relatively high concentration of exosomes albeit still contaminated with microvesicles, lipoprotein moieties, and other protein aggregates [54] (Figure 1). After this step, final exosome sedimentation occurs by spinning samples at 100,000–200,000× *g* for 2 h or at least 70 min [52]. The pellet obtained here can be resuspended in a phosphate-buffered solution (PBS) and ultracentrifuged again, which will increase the purity, but decrease the yield of the isolated exosomes [55]. The fraction of exosomes found at this level ranges from 20–250 nm [19], the size associated with exosomes, and contains RNA and microRNA species, with some of the common protein markers associated with exosomes: Flotillin-1, Alix, TSG101, CD81, CD63, and CD9 among others. However, it is important to keep in mind that some exosomal populations may not express these proteins, e.g., CD81^–^ exosomes, and/or that these markers can be found on other types of EVs as well as exosomes.

An analysis of exosome literature before 2015 reveals that 81% of studies used ultracentrifugation as their isolation technique [18,56]. However, from 2014–2017, the popularity and use of this classical technique has waned, likely due to technological advancements in exosome isolation that are less time- and labor-intensive. Among the potential disadvantages that could have precipitated this shift away from dUC is the contamination of the final product with particles of a similar size, generation of exosomal aggregates [57], as well as the duration of the procedure and the price of equipment [4,58,59]. These disadvantages, however, are counterbalanced by minute continued costs of consumables and reagents, and the reliable reproducibility of the procedure. Variations of dUC can be used to improve exosomal purity and yield. For example, density gradient ultracentrifugation separates exosomes based on size, mass, and density using a pre-constructed density gradient medium with progressively decreasing density from bottom to top of the tube [51,60,61]. In top-loading density ultracentrifugation, samples are layered on the top of the density gradient medium and vesicles’ sediment according to size and mass. Conversely, bottom-loading samples on density gradient solutions separates particles solely according to density: i.e., particles float to the point where their density is the same as the density of the medium. Thus, the gradient, in addition to the pre-existing differential speeds, facilitates the separation of smaller exosomes from the larger vesicles. A number of gradient mediums are available, however, sucrose and iodixanol (also known as OptiPrep) are commonly used for exosome isolation [54]. Density gradient can increase purity of exosomes from larger vesicles such as apoptotic bodies, but also smaller ones such as HIV-1 particles and virions. Despite these additions, some commercially available exosome isolation kits (miRCURY, ExoQuick, and Invitrogen Total Exosome Isolation Reagent) have been shown to produce a higher exosome yield of reliable quality, though less pure, when compared to dUC [18,62]. A detailed description of dUC-based exosome isolation is reviewed elsewhere [19].

### 2.2. Ultrafiltration (UF)

This technique relies on the use of membranes with specified pore diameters to isolate particles of a pre-determined size range [19,63,64]. Larger particles are eliminated first by using filters with pore diameters of 0.8 and 0.45 µm, leaving a relatively exosome-rich filtrate. Smaller vesicles are then eliminated from the filtrate by using membranes with pores smaller than the desired exosomes (0.22 and 0.1 µm) to pass into a waste eluate. The exosomes obtained are defined by a maximal and minimal size range via the first and last pore filtration membrane. This protocol can be used as a complement to ultracentrifugation to separate large microvesicles and exosomes, though it can be used as a stand-alone technique as well. Another method using nano-ultrafiltration that relies on sequential filtrations to isolate exosomes is known as cross-flow filtration or tangential-flow filtration [65]. The technique commences with a dead-end filtration of the cells and their debris along with large vesicles with a 1000 nm diameter. This is followed by tangential flow-based filtration to remove contaminants (mostly proteins) with a diameter smaller than the size cut off into a waste chamber. The filtrate, containing exosomes, is then passed repeatedly through the exclusion filter, thereby concentrating the input solution. Lastly, using a specified and consistent pore size track-etched membrane with a diameter of 50–250 nm, exosomes are further fractionated. The recovery of exosomes is dependent on the type of filter, as different membrane types and pore sizes exist: cellulose membranes with a pore size of 10 kDa have the most efficient recovery [66]. Used on its own, the exosomal preparations from UF are significantly contaminated with non-exosomal free-floating humoral peptides such as alpha-1-antitrypsin and albumin. Additionally, in comparison to dUC, UF has lower exosome yield and purity, with poorer quality of RNA, and microRNA [67]. The lower exosome purity and yield is likely due to the interaction between vesicles and the membranes, which act as a binding surface for the exosomes and proteins in the solution, creating aggregates and effectively blocking the pores. This reduces the efficiency of the UF method, decreasing both purity and yield of isolated exosomes. In contrast, SEC does not suffer from this blocking difficulty [68,69]. The advantages of UF are that it is comparatively less time- and labor-intensive and does not require the use of expensive equipment. A comprehensive overview of UF-based exosome isolation can be read here [19].

### 2.3. Poly-Ethylene Glycol (PEG)-Based Precipitation

This technique uses an aqueous PEG solution to wrap exosomes [70], facilitating the formation of exosome aggregates that can then be precipitated by low-speed centrifugation at 1500× *g* [19]. The isolated exosomal size range is in line with other methods such as dUC, however, due to co-precipitation of soluble non-exosomal proteins, the purity and specificity is largely lost. Indeed, in addition to exosomes, non-exosomal proteins, immunoglobulins, viral particles, immune complexes, and other contaminants are found in the final pellet from a PEG-based exosome isolation procedure [19,51]. Immunoprecipitating exosomes using exosome-specific markers such as CD9 [51] or other tetraspanins from the pellet obtained from a PEG-based precipitation can circumvent the lack of purity in the exosomal preparations, but generally leads to a “biased” isolation, e.g., isolating the CD9^+^ exosomal population while excluding the CD9^–^ exosomes. Overall, this method results in a high yield, but low-quality exosome isolation due to its non-specific mechanism. However, combined with an immunoprecipitation assay, it can yield immune-marker-based pure exosomal fractions. Indeed, combining PEG-based isolation with other techniques of exosomal enrichment is a viable strategy to avoid the drawbacks associated with use of PEG alone. The advantages are many: users can process many samples simultaneously, with ease, faster, and at a relatively low cost without damage to the exosomes. The drawback is the contamination of the final exosome pellet, which limits further analysis of exosomes via -omics-based assays. Nevertheless, this super-hydrophilic polymer is efficient in clinical research settings [70], which in combination with its other advantages makes it an attractive tool for crude and fast exosome extraction and analysis.

### 2.4. Immunoaffinity Capture

The immunoaffinity capture method relies on the separation of specific exosomes based on the expression of surface proteins. It commonly uses antibodies against specific exosome surface markers, specifically the tetraspanins: CD9, CD63, and CD81. Isolation of exosomes by immunoaffinity capture can be achieved by incubating the sample with the magnetic beads [71] or gold-loaded ferric oxide nanocubes [72], which are coated with antibodies against the surface proteins. Other affinity methods use markers from parent cells such as chondroitin sulfate peptidoglycan 4 [73], epithelial cellular adhesion molecule (EPCAM) [74], or exosome-binding molecules such as heat shock protein [75] and heparin [76]. Immunoaffinity is commonly used as an additional step combined with dUC to increase the purity of isolated exosomes. The main disadvantage associated with this methodology is the user selecting for a subset of marker-specific vesicles that may not reflect all exosomes. While it reduces the exosomal yield as only the antibody-recognized exosomes are captured, the isolated exosomes will be of higher purity. Furthermore, unless the antibodies can be easily removed from the vesicles post-precipitation, it can damage the integrity of exosomes [77]. The specificity and the quality of the antibody is another issue that limits the utilization of this methodology, as most antibodies commercially available for immunoprecipitation are non-specific. Overall, immunoaffinity capture is one of the most expensive methods of exosome isolation from a large sample volume, as it requires high amounts of antibody-conjugated beads, which can limit its use. Therefore, it might only be suitable for research involving small sample size, which proposes a barrier to any potential therapeutic use.

### 2.5. Microfluidics

This technique is a high-throughput method that uses microfluidic devices to isolate exosomes based on several principles including immunoaffinity, size, and density [78]. The most commonly used is the immuno-microfluidic technique, which is similar to the immunoaffinity capture isolation method. Exosomes are separated by the specific binding of antibodies immobilized on the microfluidic devices, also known as chips, to exosome markers. A common microfluidic device that has been used to isolate exosomes is ExoChip [78], with CD63 antibody. Other microfluidic devices include gold electrodes with CD9 antibody [79], graphene oxide/polydopamine (Go/PDA) nanointerface with CD81 antibody [80], and herringbone groove with CD9 antibody [81]. The advantages of this technique include efficient and fast processing, with a high level of purity of the resulting exosomal pellet. The devices are highly complex and expensive, although less expensive than the immunoaffinity capture [61]. Depending on the type of device and length of the flow channel, it can handle smaller sample volumes, as little as 10 µL, to isolate exosomes [78]. This method shares disadvantages aforementioned in the immunoaffinity capture section, in addition to the need for specialized equipment. Microfluidics is a new technology with promising prospects, but it is not yet considered a standardized method of exosome isolation. For a comprehensive review of microfluidics-based exosome isolation, please refer to this excellent review [82].

### 2.6. Size-exclusion Chromatography (SEC)

The technique uses starting biofluid as a mobile phase and a porous gel filtration polymer as the stationary phase [83]. The nature of the stationary phase allows differential elution: bigger particles elute first, followed by smaller vesicles, and then non-membrane-bound proteins. This is because the bigger the particle, the fewer pores it will be able to traverse, and thus will transverse a shorter path to the end of the column, making it elute faster in comparison to its smaller counterparts. The stationary phase or the chromatography column can be packed with a number of gel polymers including crosslinked dextrans (Sephadex), agarose (Sepharose), polyacrylamide (Biogel P), or allyldextran (Sephacryl).

The functional mechanism behind this retrieval system can be traced back to as early as 1955 when it was used for the separation of peptides from amino acids by Lindqvist and Storgårds [84,85]. This was the first use of the molecular sieve technique, known today as SEC. The dynamics behind SEC are based on size, specifically on the Stokes radii, which is synonymous with the hydrodynamic radius of the isolate in question. This radius is defined as the apparent size of the solvated/hydrated particle or vesicle [85,86]. The shape of the isolates must be taken into consideration as a factor in addition to the molecular size and weight of the isolates. If the desired particle or vesicle is not perfectly spherical, but rather has an elongated or misformed shape, it may elute at a different stage than its equally weighted spherical counterpart. Furthermore, particulate size includes the hydration shell that may surround the isolate in question. The flow rate of this fractionation mechanism also plays a role in exosomal isolation. Optimal column efficiency is found at lower linear velocites, hence decreasing the flow rate leads to a higher quality SEC assay, resulting in improved specificity, integrity, and functionality of isolated exosomes [87].

This technique was adapted into a single-step isolation system for exosomes from biological fluids in 2014 [83]. The smaller vesicles, presumably enriched with exosomes, are eluted after the larger particles have passed through. After exosomes have been captured, the last few fractions obtained are concentrated with non-exosomal proteins. Thus, SEC separates small vesicles from large vesicles, as well as removing contamination from non-exosome-bound soluble proteins, resulting in a comparatively cost-effective, pure, and intact exosome retrieval system [52]. However, it is important to note that SEC cannot differentiate between exosomes and microvesicles of the same size. Where identification of EV subtype is important to address, combining SEC with immunocapture methods is recommended.

SEC has shown to outperform other techniques in the purity of the isolated exosomes, largely by ameliorating plasma protein contamination [88]. In addition to the specificity of the technique, SEC is efficient, with a 20 min average processing time per sample [89]. This quality, however, comes at the cost of the total yield of the isolated exosomes, although SEC isolations can be scaled up. Otherwise, due to the intermediate recovery rate, a large volume of starting biofluids is required to compensate for the yield [90]. In addition to reduced total exosomal yield, the mRNA and protein yield of vesicles is also impacted [91]. Despite these issues, isolated exosomal preparations are of superior integrity [87], likely as SEC relies on the use of gravity rather than sheer force as an isolation technique. Isolated exosomes also maintain their proper vesicular characteristics. This is in stark contrast to dUC, where isolated exosomes can suffer from high shear forces that may rupture or damage the surface molecules of the isolated vesicles. Loss of integrity at the exosome surface in turn affects functionality: dUC-derived exosomes may be ineffective at binding to or activating other cells, thus interrupting the message they would otherwise communicate. SEC circumvents these pitfalls associated with what was hitherto known as the gold standard of exosome isolation.

Additionally, SEC avoids another drawback associated with dUC-based exosomal isolation: aggregation and morphological changes in the extracted vesicles. High-speed centrifugation at 100,000× *g* can form heterogeneous EV aggregates [57]. The aggregates are variable in both size and number of EVs as confirmed by cryo-TEM. Even low-speed centrifugation can create artifacts, leading to the development of multilayered microparticles (MPs). These are bilayered structures that can be found in samples centrifuged at speeds as low as 18,000× *g*, confirmed via cryo-TEM [92]. Given that dUC is currently the most widely used technique for exosome isolation, these shortcomings can contribute to erroneous conclusions related to the biophysical features and functionality of isolated exosomes. These considerations resulted in the call for the standardization of exosomal isolation techniques, and the creation of a set of guidelines to ensure quality, rigor, and reproducibility of exosome research as detailed in MISEV 2018 [18]. Preservation of exosomal integrity, functionality, and identity, despite the lower yield and some loss in purity, allows for SEC to be an ideal method for exosome isolation, especially if subsequent downstream therapeutic and biomarker discovery applications are planned [93]. It must be noted, that while SEC is a viable candidate method to isolate exosomes, direct comparative studies are required to support this conclusion.

## 3. Ideal Method for Exosome Isolation: SEC

Given the differences between the isolation techniques as mentioned above, each with respective advantages and disadvantages, an ideal method has yet to be established or agreed upon universally by researchers in the EV field. Nonetheless, a worldwide survey [56] from one hundred and ninety-six members of ISEV was collected from an online questionnaire administered via email in October 2015 [56]. The survey found that dUC was used in 85% of all cases to collect EVs from conditioned cell culture media. In contrast, UF, PEG-based precipitation, and SEC were respectively 18%, 14%, and 15% of all cases. A follow-up survey conducted in 2019 from 600 respondents by ISEV found that while dUC and density-gradient ultracentrifugation were still the most commonly used methods, the use of SEC has increased markedly, more than double its percent use from 2015 [94]. None of these techniques are exclusively optimal for exosome isolation, thus there is an understanding that an inclusive approach using multiple techniques may create the best results. Following the MISEV guidelines of 2018 [18], specificity and recovery are the main variables of each isolation technique, as summarized in Table 1. This summary table captures the evidence accumulated to date, which supports the use of SEC as an ideal exosome isolation technique compared to others.

SEC has been described as the best method for separating exosomes from most proteins, simultaneously recovering morphologically and functionally intact exosomes from plasma [95]. Exosomal preparations from SEC methodology have low levels of contaminants and co-precipitates, leading to a relatively homogenous final exosome isolation [96]. This fact has popularized the use of SEC amongst its competitors for blood-based exosome-associated biomarker discovery research [83,89]. SEC has been used successfully to isolate, purify and enrich exosomes from a variety of biological fluids including plasma [20,21,22,23,24,25], serum [26,27,28], bovine and human milk [29,30], urine [31,32,33,34], saliva [35,36], tears [35], cerebrospinal fluid [37,38], synovial fluid [39], nasal lavage [41], seminal fluid, [40] and stromal vascular fraction (SVF) from adipose tissue [42,43] (Table 2). Recent findings reported at the ISEV Virtual Conference 2020 suggest SEC is superior to other techniques in isolation of pure exosomes from human body fluids (unpublished findings). Another noteworthy advantage of SEC is that it does not require an excessive volume of sample to isolate exosomes compared to some techniques, independent of the type of sample (Table 2). It has also been used to document distinct proteomic and extracellular miRNA signatures in small exosomes isolated from conditioned media from amniotic fluid stem cells (AFSCs) [45,46], human-induced pluripotent stem cell (iPSC)-derived neurons [47], and human umbilical cord mesenchymal stromal cells (MSCs) [48], among others. An overview of the sample types that SEC has been able to isolate, purify, or enrich exosomes from is presented in Table 2. Of note is the fact that all studies included in this table have adhered to MISEV 2014/2018 guidelines to ensure high rigor and reproducibility of their work.

The recovery and purity of SEC-based exosomal isolation is especially recognizable when compared to other isolation methods, as shown in comparative studies with PEG-based isolation and dUC and UF [93,97,98]. dUC has been reported to potentially damage exosomes and can alter the exosome proteome, lipome, and/or genome [50]; UF can lead to the deformation and break-up of larger vesicles due to the pressure and contact with filter membranes [19]; PEG can co-precipitate non-EV components and alter exosomal protein signatures [93]. SEC is able to reduce or completely circumnavigate these limitations, hence, it has earned its place as a minimally invasive, rapid, and high-purity isolation technique. This is specifically an asset in blood plasma exosomal fraction isolation, as low-density lipoproteins mimic the exosomes that would otherwise be derived from the sample through dUC, which would interfere with future analysis [99]. Additionally, SEC columns are cost-effective, as one column can be washed and then reused a number of times, and can be purchased or made within the laboratory [29,89,100]. Since a single exosome isolation technique cannot reach optimal yield and purity, coupling SEC with other isolation methods including dUC, UF, or PEG-based retrieval can precipitate intact, highly purified exosomes in a reproducible manner. However, the limitations of these approaches as detailed above will then accompany the chimeric protocol [32,37,44,49,90,101,102,103].

More recently, newer techniques have been used in combination with SEC to improve exosome purity [104,105]. A technique known as dual-mode chromatography (DMC) was used successfully to reduce the contamination of lipoprotein particles (LPPs) in plasma exosome preparations [105]. This combination technique integrates two separation steps: the removal of high-density lipoproteins (HDLs) by SEC and the use of cation exchange to separate positively charged LPPs from negatively charged exosomes. Another technique combines SEC with fluorescence detection and is known as Flu-SEC or F-SEC. Here, SEC is combined with detection of fluorescently-labeled exosomes using high-performance liquid chromatography and a fluorescence detector to optimize exosome isolation [104]. Other hybrid approaches that use both SEC with PEG or dUC exist as well. It is important to keep in mind that outside of the EV research, SEC is considered to be a standard technique for purification and fractionation of peptides due to its highly reproducible and stable features [106].

Despite its prowess in recovery and specificity during exosome isolation, SEC still has its shortcomings as noted earlier in this review, and in details elsewhere [107]. In addition to not being able to distinguish same-sized exosomes from microvesicles, SEC-based exosome isolation techniques must take care to avoid denaturation of the biological targets while also controlling for unwanted electrostatic and hydrophobic interactions between the mobile phase containing the vesicles and the stationary, porous phase. The number of samples that can be processed simultaneously is another limitation associated with use of SEC. Hence, being coupled with another technique [108,109], such as ultracentrifugation, ultrafiltration, or PEG-based precipitation, may be the optimal method of isolation. SEC-coupled techniques generate a high yield of exosomes [19] that can be used for protein and RNA diagnostics as well as potentially used as a drug or drug delivery system [110,111].

## 4. Application of SEC for Isolation of Exosomes from Adipose Tissue

SEC-coupled techniques are specifically useful within the context of adipose tissue-derived exosomes [42,43,112]. Flaherty et al. [43] reported that standard isolation techniques are inefficient for extraction of exosomes from adipocytes given their high lipid content (which could affect vesicle density). This is a crucial caveat to using density-based dUC for purification of adipose-derived exosomes. A combination technique of SEC and UF was effectively utilized to isolate adipocyte-exosomes from SVF and adipose tissue-conditioned media [42,43]. Another group combined SEC with dUC to isolate adipocyte-exosomes (identified by presence of adipocyte markers) from platelet-free human plasma samples [112]. Adipose-derived exosomes enriched via SEC contain both canonical markers of exosomes, as well as adipocyte-specific proteins such as adipose triglyceride lipase (ATGL), caveolin 1 (CAV1), fatty acid-binding protein 4 (FABP4), adiponectin, perilipin, and peroxisome proliferator-activated receptor gamma (PPARγ) [43,112]. Interestingly, ATGL in adipose-derived exosomes is resistant to proteinase digestion, indicating the lipid-droplet cargo is enclosed within the vesicles [43]. However, FABP4, perilipin, and PPARγ are found as soluble proteins in circulating plasma, and FABP4 and PPARγ can be released from non-adipocytes as well [112]. As noted earlier, SEC columns [83,113] facilitate effective and reproducible exosome isolation, eliminating over 95% of non-vesicular protein from biological fluids. This is in contrast to dUC, which co-pellets soluble plasma proteins with exosomes, leading to over-estimation of adipocyte-derived exosome signatures. Thus, SEC is a superior approach to adipose-exosome isolation.

Recent papers have reported that adipose-derived exosomes constitute a relatively small proportion of the circulating plasma EVs; the majority originate from platelets, leukocytes, erythrocytes, and vascular endothelial cells [43,112]. It is important to note that a small adipose-exosome fraction does not correlate with the effect or clinical relevance of these vesicles. Seven miRNAs contained within adipose tissue-derived EVs, involved in the regulation of adipogenesis, oxidative stress, inflammation, fibrosis, and fat metabolism, were differentially expressed in lipedema patients compared to healthy controls, implicating the applicability of adipose-exosomes as biomarkers of disease [42]. Hence, using a purification technique that can use small starting volumes while maximizing yield of functionally intact exosomes free from non-vesicular protein contamination, renders the SEC-coupled approach to be the optimal method for adipose tissue-derived exosome isolation.

## 5. Conclusions

There is a lack of protocol standardization in exosome isolation methods. This void has led to the development of advanced pioneering techniques in order to optimize exosome isolation from a variety of biological fluids [114]. Currently, the optimal EV isolation method is chosen based on the amount and type of starting material (e.g., plasma, milk, cell culture media, urine, etc.), availability of specialized equipment, intended therapeutic use, route of administration, as well as desired end product (e.g., total EVs, or CD81^+^ exosomes). Considering the variable sources of exosomes, an optimized isolation method would minimize uncertainties and inconsistencies in exosomal research. Researchers need to find a balance between purity, efficiency, and downstream applications of the isolated vesicles. Through a systematic evaluation of dUC, UF, PEG-based precipitation, immunoaffinity capture, microfluidics, and SEC methods, a combined optimized protocol is advisable. Based on the efficiency, reliability, rigor, reproducibility, and ease of use, a SEC-coupled approach to exosome isolation for a high yield of homogenous, intact exosomes seems ideal. Consequentially, the data generated from such samples would expedite establishing novel diagnostic biomarkers and therapeutic drug applications of exosomes [115]. Irrespective of chosen methodology, we recommend that researchers validate the exosome isolation technique before beginning experiments, especially if using novel biofluids or samples. Thus, while SEC is an outstanding candidate method to isolate exosomes, direct comparative studies are required to support this conclusion.

## Figures and Tables

**Table 1:** Summary of different exosome isolation methods. To summarize the aforementioned isolation methods, we used a relative scaling to compare each factor listed: +++ (high), ++ (intermediate), or + (low), and in the case that grading was not applicable, we used a yes or no equivalence. *Specificity*: ability to separate exosomes; *recovery*: the amount of exosome (exosomal yield); *purity*: ability to separate exosomes with little contamination; *sample volume*: the amount of sample needed; *time*: ability to finish the processing in a short amount of time; *cost*: amount of money needed to perform the procedure; *specialized equipment*: need for expensive equipment; *complexity*: difficult to follow and need for training before use; *efficiency*: high quality sample processing; *functionality of exosomes*: ability to use the exosomes for functional studies without changing their efficacy; *scalability*: ability to process a large amount of sample without overly increasing time, cost, or personnel needed. dUC: differential ultracentrifugation, UF: ultrafiltration, PEG: poly-ethylene glycol-based precipitation, IA: immunoaffinity capture, MF: microfluidics, SEC: size-exclusion chromatography.

	dUC	UF	PEG	IA	MF	SEC
Mechanism of separation	Size, density	Size and molecular weight; through a filter membrane	Surface charge, solubility	Specific binding of antibodies to exosome markers	Immuno- affinity, density, and size	Size, shape, and molecular weight; large particles are eluted first
Specificity ^1^	++	+	+	+++	+++	++
Recovery ^1^	++	+++	+++	++	+	+++
Purity ^1^	+++	+	+	+++	+++	+++
Sample volume ^1^	++	++	+	++	+	+
Time ^1^	+++	+++	++	+++	++	+
Cost ^1^	+	++	+	+++	+++	+
Specialized equipment ^2^	++	+	+	+	++	+
Complexity ^1^	++	+	+	++	+++	+
Efficiency ^1^	++	++	++	++	+++	+++
Functionality of EVs ^2^	++	++	++	+	+	+++
Scalability ^1^	++	++	+++	+	+	+++

^1^: + (low); ++ (intermediate); +++ (high). ^2^: + (no); ++ (yes).

**Table 2:** Overview of exosome isolation from different biological sample types using size exclusion chromatography (SEC). This table summarizes the recent publications that used SEC to isolate exosomes. Shown are the various biological fluids, types of SEC columns used, starting sample volume, fractions that contained exosomes, size of isolated exosomes, and type of cargo enriched in the isolated vesicles. Some of the listed studies isolated exosomes using SEC alone or in combination with other methods. All included papers performed exosome characterization in accordance with MISEV 2018 guidelines.

Sample Type	Type of Column	Sample Volume (mL)	Fractions Used	Size of Isolated Evs	Type of Cargo	References
Plasma	Sepharose CL-2B, qEV original	1–2	4–6, 8–10, 4–7, 10–12, 7–10	20–200 nm	Proteins, miRNAs	[20,21,22,23,24,25]
Serum	qEV original, Sepharose CL-2B	0.5–1	7–9, 8–10	50–200 nm	miRNAs, proteins	[26,27,28]
Milk	qEV original, Sephacryl S-500	0.5	7–10	<200 nm	RNAs	[29,30]
Urine	qEV, Sepharose CL-4B/2B	0.5–3	8–11, 9–10, 7–10, 7–19	40–200 nm	miRNAs, proteins, RNAs	[31,32,33,34]
Saliva	miniPURE-EVs, qEV	1	7–11, 8–10	50–200 nm	miRNAs, proteins	[35,36]
CSF	Exo-spin™ mini-column, qEV single	0.1–3	5–6, 3–4	30–150 nm	Protieins	[37,38]
Synovial fluid	Sephacryl S-500 HR	–	2–4	<200 nm	Proteins	[39]
Tears	qEV	1	8–10	<200 nm	Proteins	[35]
Seminal fluid	Exo-spin™ column	1	5–9	<200 nm	–	[40]
Nasal lavage	qEV original	0.5	7–9	<200 nm	miRNAs	[41]
Stromal vascular fraction; adipose tissue	qEV70s single, Illustra Sephacryl S-1000	0.15–0.7	8–11, 8–16	50–700 nm, <250 nm	miRNAs, neutral lipids	[42,43]
Conditioned media	qEV original, Sepharose CL-2B, Sepharose CL-4B	0.5–1.5	3–7, 7–9, 7–10, 6–12	30–200 nm	mRNAs, proteins, miRNAs	[41,44,45,46,47,48,49]

**Table 3:** Factors that affect differential ultracentrifugation (dUC)-based exosome isolation. The efficiency of exosome isolation using differential ultracentrifugation is governed by four main factors: acceleration, the type of rotor in which the samples are placed, the viscosity of the solution in question, and finally the time needed to create the desired pellet [53]. This table summarizes each variable and considerations to keep in mind when isolating exosomes using dUC.

**Acceleration (*g*)**	The acceleration of the centrifuge, also known as the *g* force, refers to the speed and determines the separation efficiency.
**Rotor (*k*)**	The *k*-factor represents the relative pelleting efficiency of a rotor at maximum speed. The lower the *k* factor, the better the pelleting efficiency of the rotor, and the shorter the centrifugation time. The pelleting time (*T*) is determined by the equation *T* = *k*/s, where *T* is the time in hours required for centrifugation, *s* is the sedimentation coefficient in Svedberg units, and *k* is the *k*-factor. Sedimentation coefficients depend on the size and shape of the vesicle being isolated, and the viscosity of the sample media. The smaller the *s*, the longer it takes to pellet the particle. There are two types of rotors that are commonly used for exosome isolation: swinging bucket (SW) and fixed-angle (FA) rotors, principally differing in sedimentation efficiency. A SW rotor stands out horizontally during centrifugation, and thus has a larger sedimentation path than FA rotors. While this lowers the pelleting efficiency of SW rotors (higher *k* value) resulting in lower yield, SW rotors have better resolution, i.e., they can separate vesicles with small differences in size more effectively than FA rotors.
**Viscosity**	Reducing viscosity of the sample increases the efficiency of isolation, as the higher the viscosity, the more difficult it would be for the exosomes to travel through the sample and pellet.
**Time**	The amount of time a biological fluid is centrifuged is determined by the viscosity, rotor g value, and desired purity of the exosomal fraction. The duration can be extended to yield greater quantities of exosome-based contents such as protein and RNA, though this is limited by the possibility of condensing the pellet to such an extreme that they aggregate, making them hard to resuspend and it may thus interfere with the functional integrity of the final product. Longer time of centrifugation also co-precipitates non-exosomal proteins and reduces purity of the end product.

## Abbreviations

EV	Extracellular vesicles
sEVs	Small EVs
m/lEVs	Medium/large EVs
dUC	Differential ultracentrifugation
SEC	Size-exclusion chromatography
UF	Ultrafiltration
PEG	Poly-ethylene glycol
MVB	Multivesicular bodies
ESCRT	endosomal sorting complexes required for transport
NSF	N-ethylmaleimide sensitive factor
SNARE	soluble N-ethylmaleimide sensitive factor (NSF) attachment protein receptor
ISEV	International Society for Extracellular Vesicles
MISEV	Minimal Information for Studies of Extracellular Vesicles
PBS	phosphate-buffered solution
EPCAM	epithelial cellular adhesion molecule
Go/PDA	graphene oxide/polydopamine
iPSC	induced pluripotent stem cell
SVF	stromal vascular fraction
DMC	dual-mode chromatography
LPPs	lipoproteins particles
HDLs	high-density lipoproteins
EXO	exosomes
MV	microvesicles
AB	apoptotic bodies
AFSCs	amniotic fluid stem cells
MSCs	mesenchymal stromal cells
SVF	stromal vascular fraction
ATGL	adipose triglyceride lipase
PPARγ	peroxisome proliferator-activated receptor gamma
FABP4	fatty acid binding protein 4
CAV1	caveolin 1
